# Reflections on Applying Anti-Racist Pedagogy to Teaching About Aging

**DOI:** 10.1093/geroni/igab046.473

**Published:** 2021-12-17

**Authors:** Rona Karasik, Kyoko Kishimoto

**Affiliations:** Saint Cloud State University, SAINT CLOUD, Minnesota, United States

## Abstract

Racial disparities are well documented in all facets of aging (e.g., health, housing, retirement). Helping gerontology students to recognize racial inequity, however, is only the first step toward effecting change. Empowering students to investigate root causes of these disparities (e.g., power relations) is essential in order to begin to identify ways to dismantle institutional racism and create systems that are more equitable. With its attention to historical/political context and fostering skills for critical analyses and social change, anti-racist pedagogy offers educators a framework from which to introduce and explore these issues (Karasik & Kishimoto, 2020). This session will consider how anti-racist pedagogy may be applied within the gerontological curriculum using examples of teaching about (1) retirement and (2) housing for older adults. Associated challenges such as finding and preparing appropriate material, as well as engaging students and faculty in work they are unfamiliar and/or uncomfortable with will also be discussed.

